# Can the ChatGPT and other large language models with internet-connected database solve the questions and concerns of patient with prostate cancer and help democratize medical knowledge?

**DOI:** 10.1186/s12967-023-04123-5

**Published:** 2023-04-19

**Authors:** Lingxuan Zhu, Weiming Mou, Rui Chen

**Affiliations:** 1 Department of Urology, Renji Hospital, Shanghai Jiao Tong University School of Medicine, Shanghai, 200127 China; 2 The First Clinical Medical School, Southern Medical University, 1023 Shatai South Road, Guangzhou, 510515 Guangdong China

**To the editor,**

Large language models (LLMs) represented by ChatGPT have shown promising potential in the field of medicine [1, 2]. However, it should be noted that the answers provided by ChatGPT may contain errors [3]. In addition, other companies have launched internet-connected LLMs that can access the latest data, potentially outperforming ChatGPT which was trained on pre-September 2021 data. Prostate cancer(PCa) is the second-most common type of cancer in men globally, with a relatively long survival time compared with other cancer types [4]. Taking PCa as an example, we evaluated whether these LLMs could provide correct and useful information on common problems related to PCa and provide appropriate humanistic care, thus contributing to the democratization of medical knowledge.

We designed 22 questions based on patient education guidelines (CDC and UpToDate) and our own clinical experience, covering screening, prevention, treatment options, and postoperative complications (Table 1). The questions ranged from basic to advanced knowledge of PCa. A total of five state-of-the-art LLMs were included, including ChatGPT (Free and Plus version), YouChat, NeevaAI, Perplexity (concise and detailed model), and Chatsonic. The quality of the answers was primarily evaluated based on their accuracy, comprehensiveness, patient readability, humanistic care and stability.

**Table 1:** Questions and corresponding difficulty levels used to test the performance of LLMs

No.	Questions	Difficulty level
1	What is prostate cancer?	Basic
2	What are the symptoms of prostate cancer?	Basic
3	How can I prevent from prostate cancer?	Basic
4	Who is at risk of prostate cancer?	Basic
5	How is prostate cancer diagnosed?	Basic
6	What is a prostate biopsy?	Basic
7	How is prostate cancer treated?	Basic
8	How long can I live if I have prostate cancer?	Basic
9	How often do I need get a PSA test?	Basic
10	What is prostate-specific antigen?	Basic
11	What is screening for prostate cancer?	Basic
12	Should I get screened for prostate cancer?	Basic
13	My father had prostate cancer. Will I have prostate cancer too?	Hard
14	I have a high PSA level. Do I have prostate cancer?	Hard
15	What does a PSA level of 4 mean?	Hard
16	What does a PSA level of 10 mean?	Hard
17	What does a PSA level of 20 mean?	Hard
18	The doctor said my prostate is totally removed by surgery. Why my PSA is still high after surgery?	Hard
19	I have localized prostate cancer. Which is better, the radiation therapy or the surgery?	Hard
20	Should I have robotic surgery or laparoscopic surgery if I have prostate cancer?	Hard
21	What is the best medicine for Castration-resistant prostate cancer?	Hard
22	Which is better for prostate cancer? Apalutamide or Enzalutamide?	Hard

The accuracy of most LLMs’ responses was above 90%, except for NeevaAI and Chatsonic (Fig. 1A). For basic information questions with definite answers, most LLMs could achieve a high accuracy. Nevertheless, the accuracy decreased in questions associated with specific scenario, or in questions that involved summary and analysis (e.g., Why the PSA is still high after surgery?). Among these LLMs, ChatGPT had the highest accuracy rate, and the free version of ChatGPT was slightly better than the paid version.

**Fig. 1:**
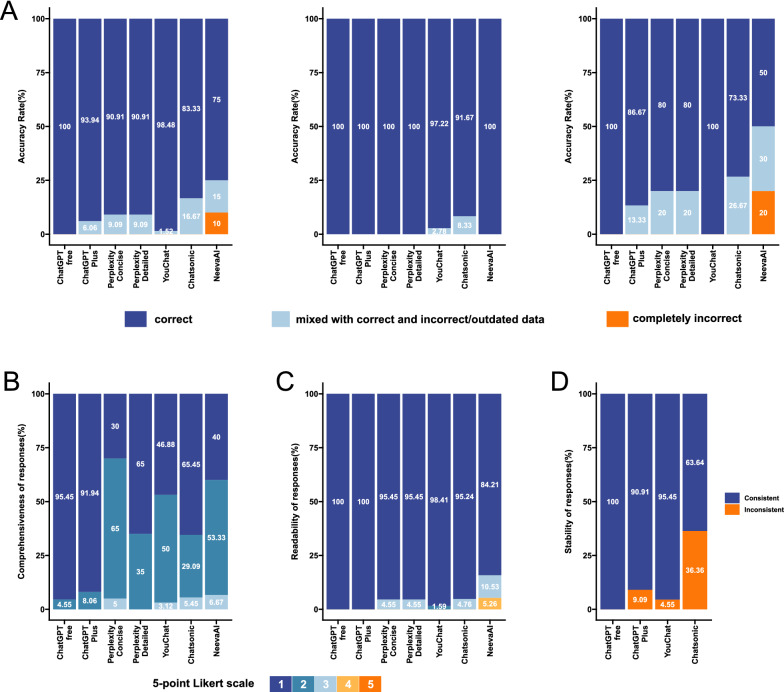
The performance of several large language models (LLMs) in answering different questions. All responses were generated and recorded on February 19, 2023. Three experienced urologists worked together to complete the ratings. **A** Accuracy of responses. Using a 3-point scale: 1 for correct, 2 for mixed with correct and incorrect/outdated data, and 3 for completely incorrect. From left to right, the performance in all questions, the performance in basic questions, and the performance in difficult questions. **B** The comprehensiveness of correctly answered responses. A 5-point Likert scale is used, with 1 representing “very comprehensive” and 5 representing “very Inadequate”. **C** Readability of answers. A 5-point Likert scale is used, with 1 representing “very easy to understand” and 5 representing “very difficult to understand”. **D** Stability of responses. Judged based on whether the model’s accuracy is consistent across different responses to the same question. Except for NeevaAI and Perplexity, the other models generated different responses each time, so we generated three responses for each question in these models to examine the stability of the models

Evaluations of comprehensiveness show that LLMs performs well in answering most questions (Fig. 1B). For example, they can effectively highlight different PSA level significance, remind patients that PSA is not the final diagnostic test, and suggest further examination. They can also compare treatment options in detail, outlining the pros and cons, and provide helpful references for patients to make informed decisions. In addition, it is commendable that most responses point out the need for patients to consult their doctors for more advice. The readability of responses from most LLMs, except NeevaAI, was satisfactory (Fig. 1C). We believe that patients can understand the information conveyed in LLMs’ responses in most cases. All LLMs could provide humanistic care when discussing expected lifespan, informing patients about the relatively long survival time of PCa, which eased anxiety. However, they did not exhibit humanistic care when answering other inquiries. LLMs’ responses were generally stable, but inconsistent outcomes were detected in some instances (Fig. 1D).

We then analyzed the reasons for the poor performance of LLMs in some responses. The most common issue was the mixture of outdated or incorrect information in the answers, including claims that open surgery is a more common choice for prostate cancer radical prostatectomy than robot-assisted surgery [5], and inaccurate responses regarding the approved indications when comparing apalutamide and enzalutamide. Inadequate comprehensiveness was mainly due to lack of specific details or omission of key points. For instance, Perplexity missed screening as an important measure in preventing PCa. Regarding the frequency of PSA testing, some answers only recommended a case-by-case approach, without specifying testing frequency for different age groups. LLMs sometimes misunderstand background information and provide inaccurate answers, such as mechanically suggesting that “PSA testing is not the final diagnostic test for PCa,” but monitoring PSA after prostatectomy is clearly not for the purpose of diagnosing PCa. It must be noted that some AI models based on search engines such as NeevaAI tend to simply provide the content of literature without summarizing and explaining, leading to poor readability. While we anticipated that the internet-connected LLMs would surpass ChatGPT, they failed to do so. This suggests that model training may be more important than real-time internet-connection.

Although not yet perfect, LLMs can provide correct answers to basic questions that PCa patients are concerned about and can analyze specific situations to a certain extent. LLMs have the potential to be applied in patient education and consultation, providing patient-friendly information to help them understand their medical conditions and treatment options, enabling shared decision-making. More importantly, LLMs can help democratize medical knowledge, providing timely access to accurate medical information regardless of geographic or socioeconomic status. This is especially important for underserved populations in medical deserts, and those facing longer waiting times for medical care during the pandemics like COVID-19. We believe that LLMs have unlimited potential with the rapid development of AI.

However, current LLMs are not yet capable of completely replace doctors, as they may contain errors or omit key points in responses, still have significant shortcomings in analyzing questions in specific contexts and cannot ask patients additional questions to gather more information. Moreover, they still cannot comfort patients like humans.

## Data Availability

The data that support the findings of this study are available on request from the corresponding author upon reasonable request.
